# Joint power, joint work and lower limb muscle activity for transitions between level walking and stair ambulation at three inclinations

**DOI:** 10.1371/journal.pone.0294161

**Published:** 2023-11-16

**Authors:** Martin Grimmer, Julian Zeiss, Florian Weigand, Guoping Zhao

**Affiliations:** 1 Institute for Sports Science, Technical University of Darmstadt, Hesse, Darmstadt, Germany; 2 Institute of Automatic Control and Mechatronics, Technical University of Darmstadt, Hesse, Darmstadt, Germany; Rosalind Franklin University of Medicine and Science Doctor William M Scholl College of Podiatric Medicine, UNITED STATES

## Abstract

To enhance human mobility, training interventions and assistive lower limb wearable robotic designs must draw insights from movement tasks from daily life. This study aimed to analyze joint peak power, limb and joint work, and muscle activity of the lower limb during a series of stair ambulation conditions. We recruited 12 subjects (25.4±4.5 yrs, 180.1±4.6 cm, 74.6±7.9 kg) and studied steady gait and gait transitions between level walking, stair ascent and stair descent for three staircase inclinations (low 19°, normal 30.4°, high 39.6°). Our analysis revealed that joint peak power, limb and joint work, and muscle activity increased significantly compared to level walking and with increasing stair inclination for most of the conditions analyzed. Transition strides had no increased requirements compared to the maxima found for steady level walking and steady stair ambulation. Stair ascent required increased lower limb joint positive peak power and work, while stair descent required increased lower limb joint negative peak power and work compared to level walking. The most challenging condition was high stair inclination, which required approximately thirteen times the total lower limb joint positive and negative net work during ascent and descent, respectively. These findings suggest that training interventions and lower limb wearable robotic designs must consider the major increases in lower limb joint and muscle effort during stair ambulation, with specific attention to the demands of ascent and descent, to effectively improve human mobility.

## Introduction

Humans utilize muscles to generate the joint power required for various movements. Reduction in joint power due to insufficient muscular capabilities, a consequence of aging or disease [1], can lead to impaired performance of power-demanding daily tasks such as ascending stairs [2–9]. To overcome these limitations, physical training has been used to improve muscle strength and thereby improve functional outcomes, such as gait speed [10–12] and postural stability [13]. In addition to physical training, wearable robotics, such as lower limb exoskeletons [14–22], could be beneficial to improve functional capacity by applying additional power to compensate for limited muscular capabilities [1]. While functional benefits of exoskeleton assistance have been shown to improve in recent years, they are still limited [15, 20] due to aspects of design [21, 22] and control [14, 20, 23]. Furthermore, most exoskeletons are designed to provide assistance only for level walking [15].

To improve training interventions and to further advance robotic exoskeleton design and control, it is critical to understand the contribution of lower limb muscles and joints to generate the movement tasks of interest. Studying particularly demanding daily tasks such as stair ascent, descent and transitions to these gaits is necessary as muscle and joint effort may be increased compared to level walking. Joint peak power can be analyzed to provide an understanding of the maximum required power output and timing; joint and limb work to provide an understanding of the energy injection, dissipation and storage and return [24]; and muscle activity (EMG) to provide an understanding of the involvement of muscles within the movement task. Previous work found that with increasing stair inclination lower limb positive peak power will increase for the hip, knee and ankle in stair ascent and for the knee and ankle in stair descent [5]. In addition, knee extensors and flexors contribute to positive work during stair ascent though they primarily contribute to negative work during level walking in order to absorb energy and decelerate segments [6]. Further, the knee extensors and ankle plantar flexors have been found to provide significant energy absorption during stair descent [9].

When summarizing available literature on the energetics and the EMG of stair ambulation, there exists no single comprehensive study analyzing the changes in effort for lower limb joints and muscles in steady level walking, stair ascent and descent at different inclinations, and the four possible transitions [4] between these gaits. Previous works that include stair ambulation energetics either analyzed not all gaits [6, 9, 26], just a single inclination [6, 9, 25, 26], no joint peak power [9, 25] or only some peak power values [5], no joint work [5, 6, 9, 26] or only net joint work [26], no limb work [5, 6, 9, 26] and no [5, 9, 25, 26] or just limited [6] EMG data was analyzed. None of the studies analyzed the transition strides in between level walking and stair ambulation [5, 6, 9, 25, 26]. In contrast to these previous studies that have concentrated solely on their individual research inquiries, a thorough examination of the combined joint and muscle effort across a range of interconnected gait conditions would provide an enhanced and more holistic perspective. This broader analysis benefits from the use of consistent reference points, such as subject groups or walking speeds, as well as the consistent extraction of data, data features and normalization techniques.

Therefore, this study aimed to comprehensively investigate changes in lower limb effort in between level walking, stair ascent and descent, and transitions from level walking to stair ascent and descent, and from stair ascent and descent to level walking for three stair inclinations (low, normal and high). In particular, we analyze the lower limb joint absolute peak power, limb and joint positive, negative and net work and the mean average value (MAV) of the EMG of lower limb muscles. We hypothesize that, compared to level walking, stair ascent and descent require more joint peak power and increased lower limb muscle activity. Further, we expect stair ascent to require more positive work as lifting the center of mass will require energy injection, and we expect stair descent to require more negative work as lowering the center of mass will require energy dissipation. In line with these expectations, we predict increases in joint peak power, work and EMG for greater stair inclinations. In addition, we expect transition strides between level walking and stair ambulation to not require an increased effort compared to steady stair ambulation, as lifting the human body against gravity requires the most effort and stair transitions only involve passing a single stair height, while steady stair ambulation entails passing two stair heights.

## Materials and methods

The present study utilizes the complete dataset obtained from an experiment conducted at the Locomotion Laboratory at TU Darmstadt during 2018 [27]. A subset of this dataset was previously used to investigate the timing of stair transitions during the transition between level walking and stair ambulation. The experimental setup and protocol, and kinematic and kinetic data processing have been described in detail in the related publication [4]. Here, we provide a summary of the key aspects of the study outlined in [4] and offer additional background information on the data, as well as novel analyses not included in the previous work.

### Subject information

This study analyzed barefoot walking kinematics, kinetics and EMG from 12 male subjects (25.4±4.5 yrs, 180.1±4.6 cm, 74.6±7.9 kg) who reported no gait-related impairments. The research protocol was approved by the institutional review board of the TU Darmstadt, and all subjects provided written informed consent in accordance with the Declaration of Helsinki.

### Experimental setup

**Fig 1:**
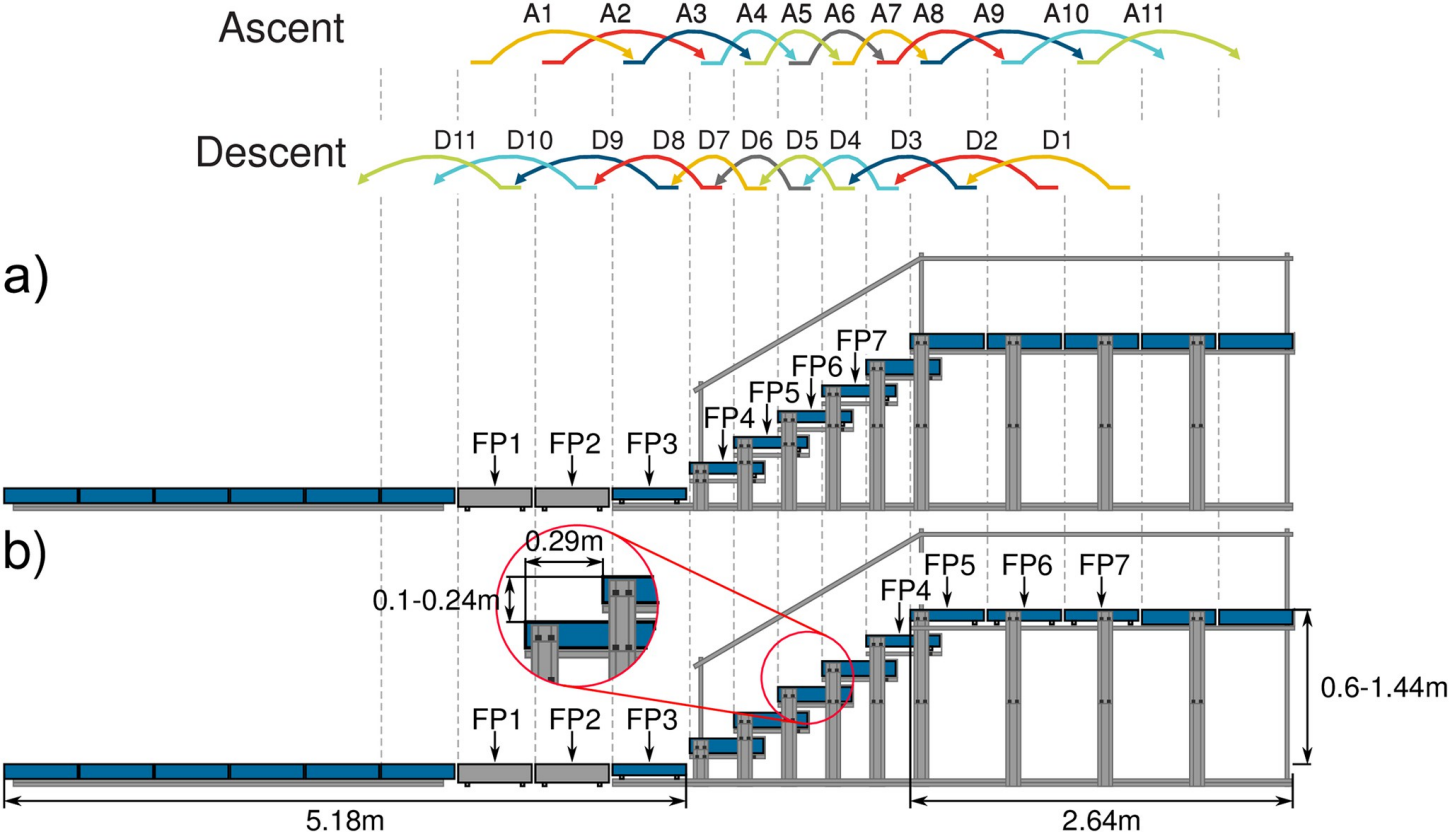
Stair and force plate configuration. Two different configurations of force plates (FP) were used to investigate four types of transitions between level walking and stair ambulation. Setup a) covers the stair transition at the lower end of the stairs and setup b) covers the stair transition at the upper end. The data from both setups was used in combination to cover strides A1 to A11 of stair ascent and strides D1 to D11 of stair descent.

Seven force plates (Kistler, Switzerland) were positioned in two configurations (setup a and b, due to a limited number of force plates available to cover the entire setup) to measure three-dimensional ground reaction forces (GRF) at 1000 Hz for a walking track that includes a staircase (Fig 1). To ensure GRF measurement from each leg when approaching and leaving the staircase, force plate positions and starting locations were arranged specifically for each subject prior to the experiment. A 3D motion capture system (Oqus, Qualisys, Sweden) recorded kinematics of 28 reflective markers at 180 Hz (see appendix of [4] for marker positions). EMG (Trigno Avanti, Delsys, Natick, MA, US) was recorded at 1111 Hz for the rectus femoris (RF), vastus lateralis (VL), biceps femoris (BF), gastrocnemius lateralis (GAS), soleus (SOL) and tibialis anterior (TA) of each lower limb. The selected frequencies for data acquisition systems were the maximum possible with the given hardware setup. All measurement frequencies were more than twice the expected maximum signal frequencies (GRF [28, 29], kinematics [30], EMG [31]) and are reasonable based on the Nyquist–Shannon sampling theorem. The wireless EMG sensors were positioned according to the Surface Electromyography for the Non-Invasive Assessment of Muscles project (SENIAM, seniam.org) guidelines. Prior to placing the sensors, hair was shaved off the skin and the skin was cleaned with alcohol. To reduce the chance of sensors loosening due to sensor movement and sweat, sensors were additionally affixed with adhesive non-woven fabric tape (Rudavlies). All measurement systems were synchronized using a hardware-based trigger signal initiated by the force plate software (Bioware, Kistler, Switzerland). The stair inclination was selected based on DIN 18065 [32]. While the stair tread depth was fixed to 0.29 m, the stair riser height was adjusted between 0.1 m (low), 0.17 m (normal) and 0.24 m (high), resulting in staircase inclinations of 19°, 30.4° and 39.6°, respectively. The normal stair height falls within the DIN 18065, and the low and high stair heights were selected to be slightly lower and higher, respectively, than the normal stair height. The strides were identified and labeled based on the force plate layout and the stair ambulation condition. Specifically, the stride that contacted the first force plate along the track (FP1) was designated as A1 for stair ascent and D1 (corresponding to FP7 from setup b) for stair descent. The labeling of strides continued with each additional stride, using labels A2 through A11 for stair ascent and D2 through D11 for stair descent (Fig 1).

### Experimental protocol

After familiarization, based on [33], ten walking trials were performed for each force plate setup at each stair height. Thus, each subject performed a total of 60 trials at their preferred walking velocity, with each trial consisting of both stair ascent and descent. Stair height was changed based on the previous subject’s last setup, in order of high-normal-low and low-normal-high. Breaks of at least one minute were required between trials to store data. Breaks of at least 30 minutes were required to change stair height. Subjects walked barefoot and wore tight swimming trunks.

### Data processing

In addition to the methods outlined in [4], we calculated positive and negative joint work by numerically integrating the hip, knee, and ankle power for each stride. Net joint work is obtained by integrating power over time. Limb work is the sum of the respective hip, knee and ankle work. The absolute maxima were extracted from the individual power curves to determine the peak power specific to each joint.

To analyze the muscle activity (EMG) throughout the gait cycle, the signal offset was subtracted, signals were bandpass filtered (zero-lag, 20 Hz to 450 Hz, fourth-order Butterworth), rectified and low-pass filtered (zero-lag, 6 Hz, second-order Butterworth) [31]. Following, the EMG signals were extracted for each stride primarily using the ground reaction forces as described in [4]. For each stride and muscle, the mean average value (MAV) of the EMG was determined.

Due to loose sensors, the EMG data from some conditions was excluded entirely from the analysis (TA for all inclinations for two subjects, BF for low, VL for high and GAS for high stair inclination for one subject each). In addition, EMG data from outlier strides (data that differs greatly from all the rest due to unknown reason) was excluded from further analysis. Outlier strides were identified using a subject-specific mean MAV of all conditions plus five times its standard deviation as the cutoff (with one limit determined for each muscle). Approximately 4% of the EMG data was excluded in total.

For each stride (A1 to A11 and D1 to D11) the subject mean of each EMG was determined. From the individual subject means of the EMG from level walking for the strides A1 (right limb) and D11 (left limb) the maxima throughout the stride were extracted for a separate normalization of the right and left limb. The maxima were used to calculate a subject- and limb-specific normalization factor, which normalizes the maxima to be 100%. For normalization, the subject- and limb-specific factors were multiplied by the subject means and the MAVs of the EMG. Joint work and peak power were normalized to body mass. The stride-specific subject means for peak power, joint and limb work, and EMG were computed based on 10 trials per stride, except for strides A1 and D9 through D11, which were measured in both setups, resulting in 20 trials per subject. Grand means and standard deviations were determined based on the subject means.

### Statistics

Changes from level walking (A1, normal) to stair ascent (A6) and level walking (A1, normal) to stair descent (D6) were statistically analyzed using a repeated measures ANOVA. The Mauchly test was used to test for sphericity. If required, a correction was performed using the Greenhouse-Geisser adjustment. Comparisons were performed for each muscle, the joint peak power and the joint positive and negative work (15 parameters in total). A post hoc analysis (paired-sample t-test) was performed to test for a significant difference between the parameters of A1 and A6 and A1 and D6. To counteract type I errors, a Holm-Bonferroni step down correction was used. However, the large number of comparisons performed (90 due to A1 to A6 and A1 to D6, for three inclinations, for 15 parameters) has a high likelihood to also cause type II errors [34]. Prior to the correction, 11 comparisons were not significant with a p-value of larger than 0.05. Following correction, 27 values were not significant at the 0.05 significance level including comparisons with a previous p-value of up to 0.0019. The effect size was determined using unbiased Cohens d [35] with a confidence interval at 95%.

## Results

**Fig 2:**
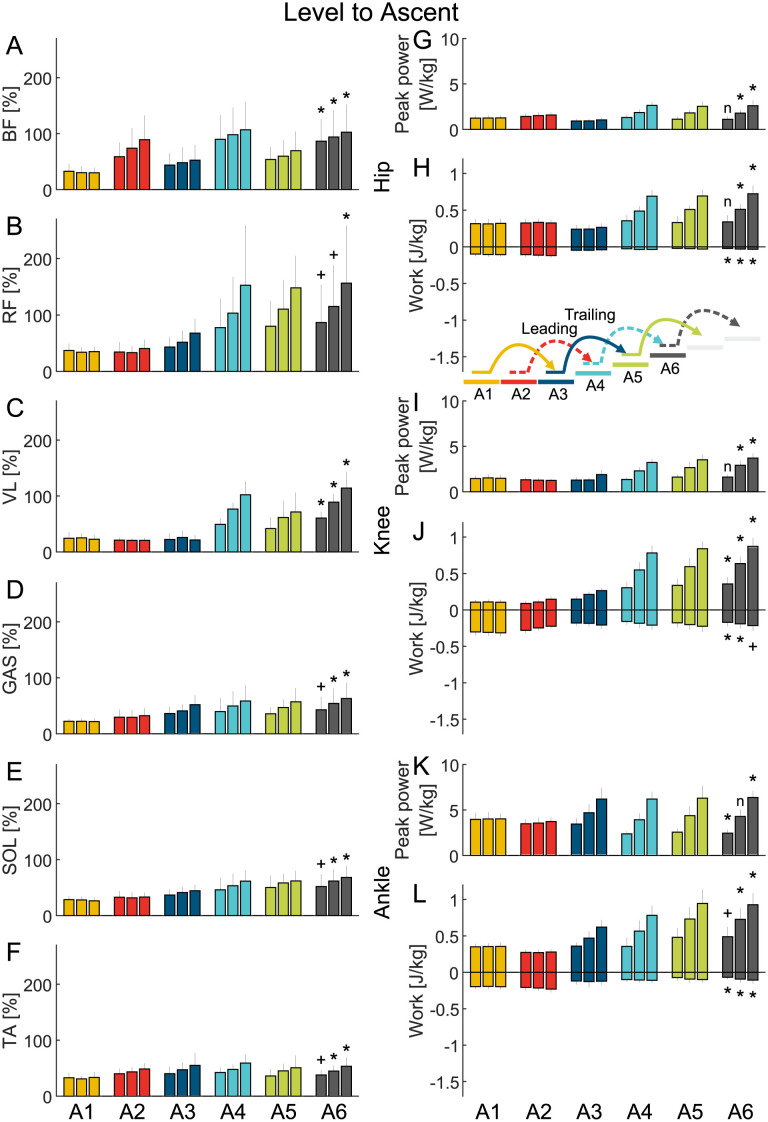
Level to ascent EMG and joint work. Grand mean average value (MAV) of the EMG (A-F) of the biceps femoris (BF), rectus femoris (RF), vastus lateralis (VL), gastrocnemius (GAS), soleus (SOL) and tibialis anterior (TA) and grand mean of the hip (G and H), knee (I and J) and ankle (K and L) peak power and positive and negative work. The data is shown for six strides (A1-A6) and three stair inclinations (data bars from left to right: low, normal and high) and includes the standard deviation. A plus (+) indicates a significant difference between the level walking stride A1 and the stair ascent stride A6. A star (*) indicates a significant difference between the level walking stride A1 and the stair ascent stride A6 after type I error correction. An n indicates that there was no significant difference.

**Fig 3:**
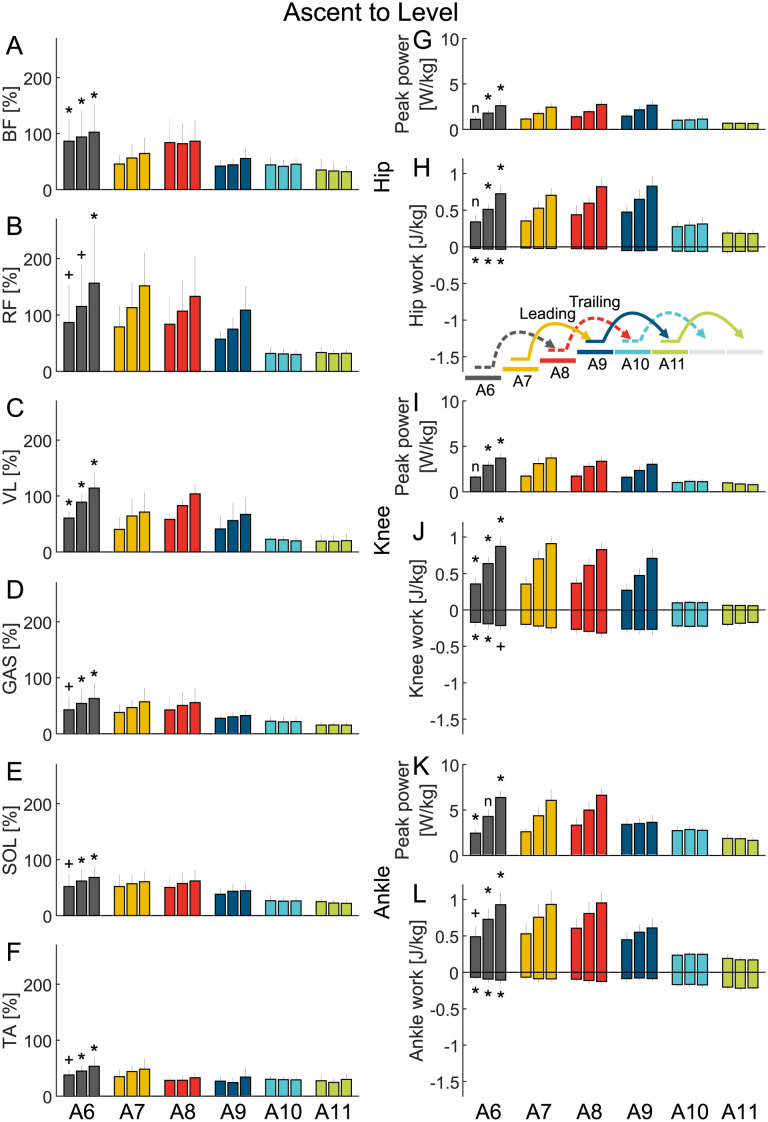
Ascent to level EMG and joint work. Grand mean average value (MAV) of the EMG (A-F) of the biceps femoris (BF), rectus femoris (RF), vastus lateralis (VL), gastrocnemius (GAS), soleus (SOL), and tibialis anterior (TA) and grand mean of the hip (G and H), knee (I and J), and ankle (K and L) peak power and positive and negative work. The data is shown for six strides (A1-A6) and three stair inclinations (low, normal, high, three close bars from left to right) and includes the standard deviation. A plus (+) indicates a significant difference between the level walking stride A1 and the stair ascent stride A6. A star (*) indicates a significant difference between the level walking stride A1 and the stair ascent stride A6 after type I error correction. An n indicates that there was no significant difference.

**Fig 4:**
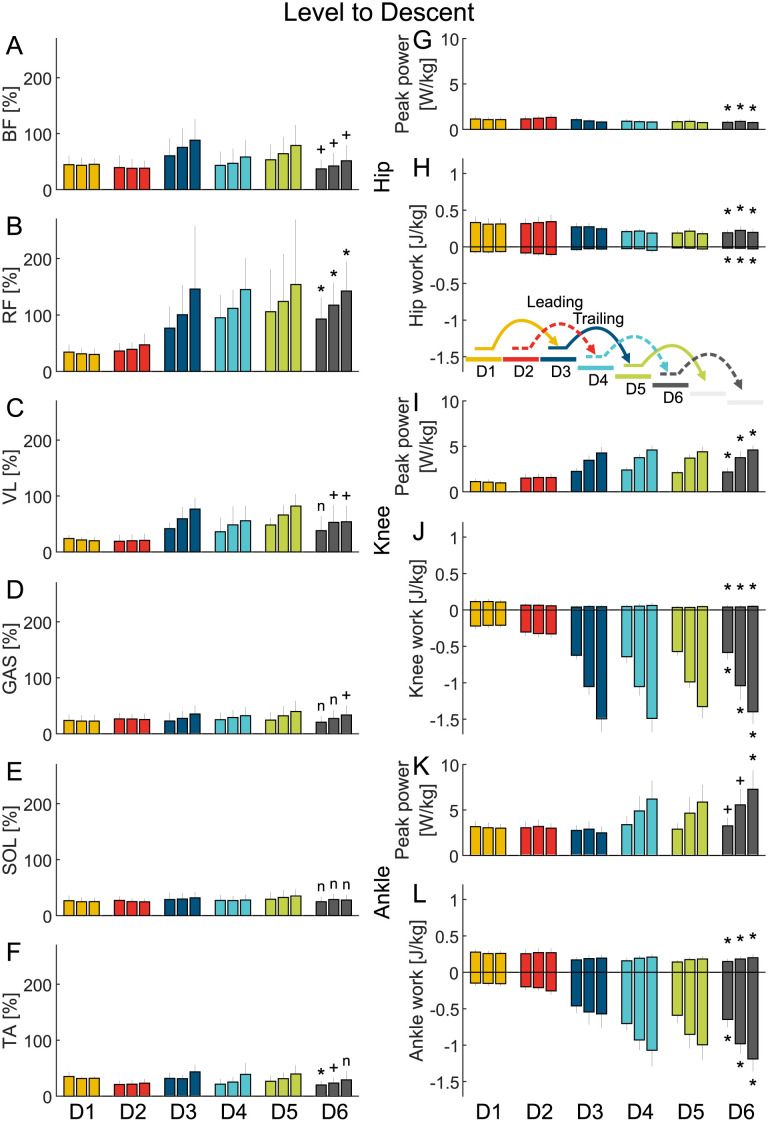
Level to descent EMG and joint work. Grand mean average value (MAV) of the EMG (A-F) of the biceps femoris (BF), rectus femoris (RF), vastus medialis (VL), gastrocnemius (GAS), soleus (SOL), and tibialis anterior (TA) and grand mean of the hip (G and H), knee (I and J), and ankle (K and L) peak power and positive and negative work. The data is shown for six strides (A1-A6) and three stair inclinations (low, normal, high, three close bars from left to right) and includes the standard deviation. A plus (+) indicates a significant difference between the level walking stride A1 and the stair descent stride D6. A star (*) indicates a significant difference between the level walking stride A1 and the stair descent stride D6 after type I error correction. An n indicates that there was no significant difference.

**Fig 5:**
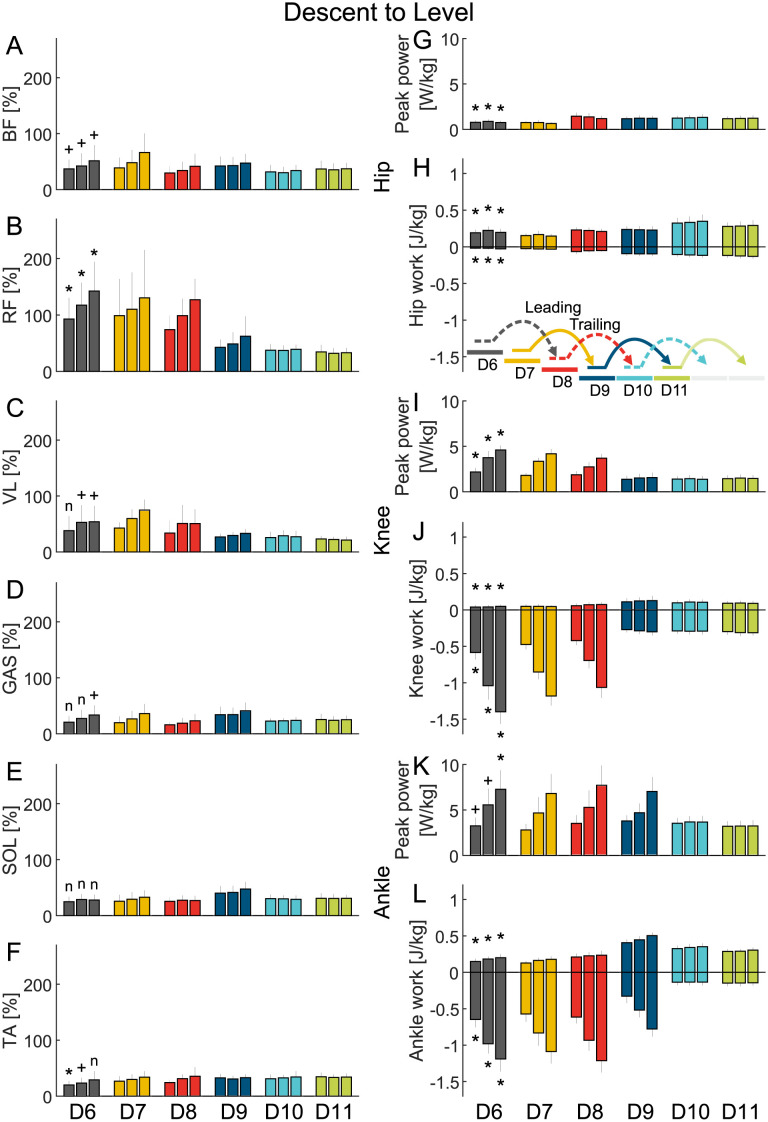
Descent to level EMG and joint work. Grand mean average value (MAV) of the EMG (A-F) of the biceps femoris (BF), rectus femoris (RF), vastus medialis (VL), gastrocnemius (GAS), soleus (SOL), and tibialis anterior (TA) and grand mean of the hip (G and H), knee (I and J), and ankle (K and L) peak power and positive and negative work. The data is shown for six strides (A1-A6) and three stair inclinations (low, normal, high, three close bars from left to right) and includes the standard deviation. A plus (+) indicates a significant difference between the level walking stride A1 and the stair descent stride D6. A star (*) indicates a significant difference between the level walking stride A1 and the stair descent stride D6 after type I error correction. An n indicates that there was no significant difference.

Our results focus on increases in joint peak power, limb and joint work, and EMG for steady stair ambulation strides (A6 and D6) and stair transitions, compared to level walking (A1). While trends can be easily observed in the respective figures (Figs 2–5), numerical results can be found in Table 1. In addition, you can find time normalized power and EMG data for each stride and condition and information about their relation in the supplementary information and figures (S1 to S6 Figs in S1 File).

**Table 1:** Overview of the peak power, positive and negative work, and EMG for strides A1, A6 and D6 from the low, normal and high stair inclination for the hip, knee, and ankle.

Variable	Stride	A1 Normal	Low	A6 Normal	High	Low	D6 Normal	High
Peak power [W/kg]	Hip	1.3±0.3	1.1±0.3	1.8±0.4	2.6±0.6	0.8±0.2	0.9±0.2	0.8±0.2
	Knee	1.5±0.4	1.6±0.3	2.9±0.5	3.7±0.5	2.2±0.4	3.8±0.7	4.6±0.5
	Ankle	4.0±0.7	2.5±0.4	4.3±0.8	6.4±0.7	3.3±0.9	5.6±1.8	7.3±2.1
Positive work [J/kg]	Hip	0.31±0.06	0.34±0.09	0.51±0.07	0.72±0.11	0.19±0.04	0.22±0.05	0.2±0.05
	Knee	0.11±0.03	0.36±0.09	0.64±0.09	0.87±0.12	0.04±0.03	0.04±0.02	0.05±0.02
	Ankle	0.35±0.05	0.49±0.14	0.73±0.15	0.93±0.16	0.15±0.04	0.18±0.03	0.2±0.05
	Limb	0.78±0.09	1.19±0.09	1.88±0.11	2.52±0.13	0.38±0.06	0.45±0.06	0.45±0.07
Negative work [J/kg]	Hip	-0.1±0.03	-0.02±0.01	-0.03±0.02	-0.03±0.02	-0.02±0.01	-0.02±0.01	-0.03±0.01
	Knee	-0.31±0.05	-0.17±0.05	-0.19±0.06	-0.21±0.07	-0.58±0.1	-1.04±0.19	-1.4±0.16
	Ankle	-0.19±0.04	-0.07±0.03	-0.09±0.05	-0.11±0.05	-0.65±0.11	-0.98±0.13	-1.19±0.17
	Limb	-0.61±0.07	-0.26±0.07	-0.31±0.08	-0.35±0.08	-1.25±0.13	-2.04±0.18	-2.62±0.16
Net work [J/kg]	Hip	0.21±0.07	0.32±0.09	0.48±0.07	0.69±0.11	0.17±0.04	0.2±0.05	0.17±0.05
	Knee	-0.21±0.04	0.19±0.08	0.45±0.07	0.66±0.13	-0.55±0.1	-1.0±0.18	-1.35±0.16
	Ankle	0.16±0.05	0.42±0.12	0.63±0.12	0.82±0.12	-0.5±0.13	-0.8±0.15	-0.99±0.18
	Limb	0.17±0.05	0.92±0.06	1.56±0.07	2.17±0.08	-0.87±0.08	-1.59±0.13	-2.17±0.13
EMG [%]	BF	30±11	86±39	94±48	103±50	37±17	42±23	51±28
	RF	34±9	87±66	115±73	156±102	93±38	117±40	142±52
	VL	25±8	61±11	89±15	114±29	38±26	53±31	54±28
	GAS	22±4	43±23	54±27	63±28	21±11	27±16	34±17
	SOL	28±6	52±22	62±21	68±20	25±9	29±10	28±9
	TA	31±5	38±9	45±10	53±16	20±7	23±9	29±16

### Steady gait and inclinations

Subjects approached the staircase with a preferred level walking velocity of 1.25±0.13 m/s (A1). Stair ascent was performed on average at 0.51±0.04 m/s (A6) and stair descent at 0.56±0.07 m/s (D6) [8].

#### Peak power

Compared to level walking (A1), significantly greater peak power was required during normal stair ascent (A6) for the hip (p=.04, d=-1.4 [-2.7 -0.6]) and knee (p = 1.4E-05, d=-2.8 [-4.6 -1.8]) and during normal stair descent (D6) for the knee (p = 5.7E-05, d=-3.5 [-5.8 -2.1]). With increasing stair inclination during stair ascent, the peak power increased for the hip, knee and ankle. With increasing stair inclination during stair descent, hip peak power remained equal while it further increased for the knee and for the ankle.

#### Work

Compared to level walking (A1), normal stair ascent (A6) showed a significant increase in positive work and a significant decrease in negative work at the hip (p = 2.5E-05, d=-2.7 [-4.4 -1.7] and p = 6.1E-04, d=-2.8 [-4.8 -1.6]), knee (p = 1.4E-05, d=-2.8 [-4.6 -1.8] and p=.001, d=-1.9 [-3.3 -1.1]) and ankle (p = 2.3E-04, d=-3.2 [-5.2 -1.9] and p=.003, d=-2.2 [-3.7 -1.2]). On the other hand, normal stair descent (D6) showed a significant decrease in positive work (p=.049, d = 1.4 [0.6 2.6]) and negative work (p = 8.1E-05, d=-3.4 [-5.5 -2]) at the hip, while negative work significantly increased at the knee (p = 4.9E-06, d = 4.9 [3.1 7.9]) and ankle (p = 1.4E-07, d = 7.4 [4.7 11.8]).

As stair inclination increased, positive work increased for all joints during stair ascent, and negative work increased for the knee and ankle. However, no changes in negative hip work were observed with increasing stair inclination.

Net limb work during level walking was slightly positive. Normal stair ascent and descent required nine times the net limb work of level walking, either as positive work during ascent or negative work during descent.

#### EMG

During level walking (A1), the EMG MAVs for the BF, RF, VL, GAS, SOL, and TA ranged between 22% and 34%. In contrast, normal stair ascent (A6) resulted in significantly increased EMG MAVs ranging from 45% to 115% except for the RF that showed a high though non-significant increase (BF p=.006 d=-1.7 [-3 -0.9], VL p = 2.3E-06 d=-5 [-8 -3.1], GAS p=.04 d=-1.6 [-2.9 -0.6], SOL p=.008 d=-2.1 [-3.6 -1], TA p=.002 d=-1.6 [-2.9 -0.9]). Only RF showed a significant increase in MAV (117%) during normal stair descent (D6) compared to level walking (p = 3.5E-04, d=-2.7 [-4.5 -1.6]).

Moreover, increasing stair inclination led to a further increase in MAVs for all analyzed muscles during stair ascent. Similarly, we observed a clear trend of increasing MAVs for all muscles, except for SOL and VL, during stair descent.

### Stair transitions

For the level to ascent, the ascent to level, and the level to descent transitions the joint work and the MAVs of the EMG changed from one to the other gait condition without increasing in amplitude compared to steady gait (Figs 2 to 4).

However, for the descent to level walking transition the positive ankle work for stair transition stride D9 nearly doubled compared to steady level walking and stair descent strides. Further, the MAV of the GAS and SOL were highest in D9 (Fig 5). Additionally, in the descent to level transition the positive hip work during D10 was found to be highest while neither of the muscles spanning the hip (BF, RF) showed a similar increase (Fig 5).

## Discussion

In this study, we aimed to investigate the changes in lower limb effort during steady gait and gait transitions between level walking and stair ambulation by analyzing the lower limb joint peak power, limb and joint work as well as the muscle activity of major lower limb muscles.

### Steady gait requirements

As we had hypothesized, our results showed significant increases in joint peak power, work and EMG activity during stair ascent and descent compared to level walking, and these values further increased with the inclination of the stairs. Previous studies have shown similar outcomes though with partially lower power amplitudes and smaller increases in peak power [5, 25, 26]. One major reason for the difference could be the reduction in stair ambulation velocity, which was approximately 20% less in ascent and 12% less in descent for [5] and 12% less in ascent and 20% less in descent for [25], compared to A6 and D6 [4]. While walking velocity was fixed by a metronome in [25], subjects were walking at their preferred velocity in [5] and this study. As our stair dimensions and subject population were similar to [5], we believe that the length of the walkways needed to accelerate and decelerate when either leaving or entering the staircase, respectively, could have contributed to a reduced preferred stair ambulation velocity.

Similar to the findings for walking uphill and downhill [36], our data clearly demonstrates that, compared to the slight positive net work contribution during level walking, stair ascent and descent require significantly increased net positive and net negative work to lift or lower the center of mass, respectively [4]. Specifically, at the high stair inclination, thirteen times higher net positive and net negative limb work is required for stair ascent and descent, respectively, compared to level walking, revealing that stair ambulation is much more demanding for muscles to perform.

In addition to the increased work, muscles also have to generate approximately double the peak power for most joints at the high inclination. While all muscles showed a significant increase in EMG for stair ascent, only the RF showed a significant increase for stair descent. We assume that this is caused by eccentric muscle behavior for energy dissipation in stair descent, which is less demanding than the concentric behavior required for energy injection in stair ascent as found in a dynamometer-based study [37]. While a direct measurement of muscle force is not feasible, we can speculate that the enhanced efficiency of eccentric muscle lengthening during stair descent enables greater forces to be generated at comparable levels of EMG for various lower-limb muscles. Consequently, this could lead to an augmented peak power output at the joint level.

Our findings indicate that not only do absolute power levels increase, but the distribution of power between joints also changes. Previous research [38] demonstrated that during level walking at a speed of 1.25 m/s, the hip generates 40% of average positive power, the knee generates 14%, and the ankle generates 46%. Our data on positive joint work during level walking (A1) confirms this relationship (41%, 14% and 45%), emphasizing the critical role of the hip and ankle in injecting energy during level walking. We observed that stair descent has a similar distribution (50%, 9% and 41%, D6, normal stair inclination), with a slight shift in contribution towards the hip. In contrast, stair ascent involves a substantial contribution of positive joint work by the knee (34%), with reduced importance for the hip (27%) and ankle (39%, A6, normal stair inclination). Our results on joint work and the significant increase in EMG activity of muscles surrounding the knee (BF, RF, VL, GAS) support the findings of [6], which suggest an increased role of the knee in achieving stair ascent.

One might expect a similar relationship during uphill walking. However, Nuckols et al. [36] found less average positive power contribution from the knee (19%) and an increased contribution from the hip (52%) during uphill walking. The slope investigated in their study was a maximum of 8.5°, which may partially explain the difference in results when compared to our study, which investigated slopes ranging from 19° to 39.6°. The knee appears to play a significant role in providing energy to lift the center of mass, while energy injection by the hip may be advantageous at higher walking velocities (1.25 m/s in [36] compared to 0.51 m/s in our study, A6) to lift and swing the leg forward as in level walking.

Negative joint work contributions during level walking were found to be 17%, 50% and 33% for the hip, knee and ankle, respectively (A1, normal). In comparison, during stair ascent (A6, normal stair inclination), the contribution of negative joint work shifted slightly from the hip to the knee (10%, 60% and 30% for hip, knee and ankle, respectively). Stair descent (D1, normal stair inclination) showed almost no negative joint work at the hip (1%), while the knee and ankle made nearly equal contributions (51% and 48%, respectively), supporting previous research on the significant energy absorption by these joints [9]. In contrast to level walking, where the hip provided negative joint work to decelerate the lower limb during late stance, stair descent required only positive work to swing the limb forward and stabilize and retract the limb after heel strike. For downhill walking, Nuckols et al. [36] found a dominant contribution of average negative power from the knee (62%) and smaller contributions from the ankle (27%) and hip (11%). We assume that the differences for the ankle between stair descent and downhill walking exist due to the manner in which the ground is approached. While in stair descent subjects touch the ground with the forefoot, in downhill walking subjects touch the ground first with the heel. Using the forefoot could allow for a larger energy dissipation at the ankle, which is reflected in the negative joint work.

The results on joint contributions in stair descent and downhill walking highlight the minor energetic role of the hip for both tasks. In addition, previous work found nearly constant hip positive peak power during stair descent [5], and we have shown that not only positive and negative peak power but also the hip work stays relatively constant for different stair inclinations. In addition, in contrast to our predictions, no negative work and less peak power were required at the hip in stair descent compared to level walking. These findings suggest that the hip does not contribute to modulating power with changing stair inclination during stair descent. Opposing hip peak power and hip work, the BF and RF, which span the hip, show increases in activity with increasing stair inclination in stair descent. These increases mainly occur within the stance phase while substantial hip power is required during the swing phase. In contrast, substantial knee power is required during stance and thus we believe that the increases seen for both muscles primarily reflect the increasing power demands of the knee when increasing stair inclination.

### Stair transition requirements

Our analysis indicates that, in most cases, stair transition strides do not require increased positive or negative joint work, peak power or muscle activity compared to steady level walking or stair ambulation. However, one exception is stride D9 during the transition from descent to level where we observed increased positive ankle work compared to level walking and stair descent. This increased work may be necessary to increase forward velocity, as has been reported in gait initiation studies [39]. In addition, MAV of the GAS and SOL were highest in D9 during this transition. This is likely due to the fact that a single stride must realize both the lowering of the foot that is typically found in stair descent and the push-off that is typically used during level walking (see S1–S3 Figs in S1 File).

Another noteworthy finding is the high positive hip work of stride D10 during the descent to level transition, which is likely associated with increased extension power realized in this stride to enhance stride length and forward velocity (see S6 Fig in S1 File). This extension power further extends the hip joint to angles that are normally achieved in level walking [4]. The BF muscle, which assists with hip extension, showed slight increases in activity during early stance in D10 compared to steady stair descent and level walking, although the MAV remained below that of stair descent and level walking (see S3 Fig in S1 File).

However, it is important to note that although D9 and D10 require the highest positive ankle and hip work in the descent to level transition, the values are still lower than those required for stair ascent.

### Applications

The comparison of the individual joint contributions from level walking versus stair ascent and descent indicates that the knee joint plays a critical role in energy provision and absorption. This is evidenced by the only significant increase in the MAV of the RF, a knee extensor, during stair ascent and descent. Thus when improving a level walking wearable robotic design, the actuator average and peak power specifications of the knee should be increased in particular to augment the level of stair ambulation assistance. However, the ankle also provided substantial joint work in stair ascent and descent. If potential lower limb wearable robotics users suffer from physiological deficits that limit the generation of joint work (e.g., due to respiratory, cardiovascular, musculoskeletal or neurological diseases [1]), external energy assistance, with a specific focus towards assisting the knee and ankle joints, may be beneficial to improve both stair ascent and descent capabilities. However, energy injection from the hip is critical during stair ascent and uphill walking [36], and this demonstrates that each lower limb joint could be considered when designing wearable robotics for daily use. In line with this, training interventions should focus on all lower limb muscles to improve the muscle average and peak power.

The results on peak power and work provide valuable insights for determining battery specifications for autonomous wearable robotics designed to assist with both level walking and stair ambulation. One crucial factor to consider when determining battery capacity is the number of strides intended per day. Elderly individuals, which are one target population for assistive wearable robotics, exhibit an average of 4,241 strides per day [40]. Of these strides, only a small amount, approximately 47 to 66 strides on average per day, are attributed to stair climbing [41, 42]. As the amount of positive hip and ankle work required for stair ascent is only double that of level walking, the daily battery requirements will not need to significantly increase. However, robotic knee designs for level walking in particular could be limited when performing stair ascent as it requires more than eight times the positive work for the knee. In addition, based on the mechanical design to perform negative work, increases of up to six times for the knee and eight times for the ankle, compared to level walking, could considerably limit runtime. To address this issue, robotic solutions could incorporate dampers [43, 44] or even energy harvesting mechanisms [45] to account for the negative work and avoid the need for increases in battery capacity.

In addition, it’s important to note that the increased power demands during stair ambulation could significantly increase the heating of all electrical components and the connected human-machine interface. However, since stair ambulation is typically performed for short intervals compared to level walking, this may be advantageous in avoiding critical heat levels. The high-intensity nature of stair ambulation, which is performed at the human physiological limit [1, 46], should also be taken into consideration when developing training interventions.

Interestingly, transitions between different gait conditions did not show increased power requirements compared to steady gait conditions. Thus, the steady-state conditions of level walking, stair ascent and stair descent can be used to determine the peak power and work requirements of lower limb joints that are necessary to perform these gaits and gait transitions when designing an assistive wearable lower limb robot.

### Limitations

It should be acknowledged that due to individual EMG normalization for the left and right limbs (from stride D11 and A1, respectively) that was based on the maxima of the individual subject means in level walking, it is possible that one limb showed a larger grand mean over time or a larger MAV than the other. Such effects could be also caused by laterality. A distinct difference was seen in the BF with larger values for the right limb. Sung and Lee reported a significant difference in muscle activity between the dominant and non-dominant limb in stair ascent, with the dominant limb showing a higher vastus medialis amplitude and earlier onset [47]. A normalization of the EMG with the use of a maximum voluntary contraction could minimize such effects. However, as we already had an extensive stair ambulation protocol, maximum voluntary contractions were not performed. In addition, we did not gather information about laterality, nor did we design the protocol to minimize effects of laterality. Furthermore, the subjects in this study were exclusively young, male adults without self-reported gait impairments. However, previous research has shown that there are significant gender-based differences in muscle activity during stair ambulation, although the magnitude of these differences was not reported in this study [47]. Additionally, approximately 4% of the EMG data had to be excluded from the analysis. It is believed that sweating, combined with the weight of the wireless sensors, caused sensors to move on the skin, resulting in poor data quality. To address this issue, the use of wireless sensors with an external communication unit could be beneficial in reducing the amount of data lost. Further, subjects walked barefoot in our study. Also while we believe the changes are small, walking with shoes will impact gait kinematics, kinetics and EMG [48]. Last, be aware that due to the type I error correction there is a high chance for type II errors [34] as 90 comparisons for significance were performed in total as described in the Statistics section.

## Conclusion

This study found that stair ambulation requires increased joint and muscle effort compared to level walking, with the effort increasing as stair inclination increases. However, there was an exception in the hip during stair descent, which showed almost no negative joint work or response to stair inclination, suggesting that the hip does not contribute to modulate stair descent energetics. The study also revealed that there were no significant increases in effort during the transition strides compared to the maxima of steady gait conditions. This suggests that transition strides may not be a significant factor with respect to energetics in the development of wearable robotic designs. Furthermore, wearable robotic designs should take into account that, compared to level walking, stair ascent requires increased lower limb joint positive work and peak power, while stair descent requires increased lower limb joint negative work and peak power. Solutions that can inject and dissipate energy are necessary to address this demand. Additionally, the energetic contribution of the knee joint increases greatly during stair ambulation compared to level walking. However, the hip, knee and ankle contribute with high energy injection to stair ascent and the knee and ankle with high energy dissipation to stair descent. Thus, all three joints could be targeted with lower limb assistive robotics or training interventions to compensate for the increased energy demand. Lastly, as walking requires a continuous load at a low to medium intensity level, professionals involved in designing robotics or training interventions should consider the short duration, highly intensive nature of stair ambulation.

## Supporting information

S1 File (PDF)

S1 Data (MAT)

S2 Data (MAT)

S3 Data (MAT)
