# Supplementary material for: Joint power, joint work and lower limb muscle activity for transitions between level walking and stair ambulation at three inclinations
Source: PLoS One. 2023 Nov 16;18(11):e0294161. doi: 10.1371/journal.pone.0294161 (PMC10653464; doi:10.1371/journal.pone.0294161)
Supplement: S1 File — (PDF) [file pone.0294161.s001.pdf]

Supporting information

Functional relation of joint power and EMG

Hip The increase in hip power during stair ascent was found to be primarily attributed to greater hip extension power from late swing through stance. The BF, a hip extensor, exhibited these increases with earlier activity in the swing phase and increased activity during most of the stance phase compared to level walking (see S1 Fig to S6 Fig).

During stair descent primarily positive work was provided by the hip. The hip flexed to allow a free swing down each stair and extended before heel strike [4]. Increases in BF activity were found for both hip flexion and extension, and activity during early hip flexion was found to be most sensitive to stair inclination.

Knee The major increase in positive power during stair ascent occurred during the first half of stance, and this was reflected in the increase in EMG of the RF and VL (see S1 Fig to S6 Fig). A further increase in knee positive power occurred in early swing, which is reflected by the increase in activity of the RF. The GAS and BF muscles, which also cross the knee joint, appear to aid with positive work for knee flexion during the late stance and early swing phases of stair ascent, as evidenced by their increased muscle activity coinciding with positive work. This trend holds true for all stair inclinations.

When descending stairs, the majority of negative work required from mid- to late stance was used to flex the knee joint, thereby lowering the center of mass [4]. This negative work can be accomplished by eccentric muscle activity of the knee extensors RF and VL, which elongate under load. Both muscles exhibited significant increases in activity during mid- to late stance compared to level walking (see S1 Fig to S3 Fig). Additionally, negative work increased during the mid-swing phase of stair descent due to increased knee flexion. This increased flexion was accomplished by the GAS and BF muscles, which exhibited increased activity during this phase.

Ankle During stair ascent, ankle power increased during the mid-stance and push-off phases, which can be attributed to increased activity in both the plantarflexor muscles GAS and SOL (see S1 Fig to S6 Fig).

In contrast, during stair descent, power increases mainly occurred in the early stance phase (see S4 Fig to S6 Fig). In level walking, the TA controlled eccentric plantarflexion after heel strike, contributing to negative ankle work. However, during stair descent, the TA activity in early stance was lower than that in level walking where the former involved a forefoot strike rather than a heel strike. Thus, controlled eccentric dorsiflexion was required to lower the whole foot towards the ground during early stance, which was realized by greater activity of the calf muscles GAS and SOL (see S1 Fig to S3 Fig).

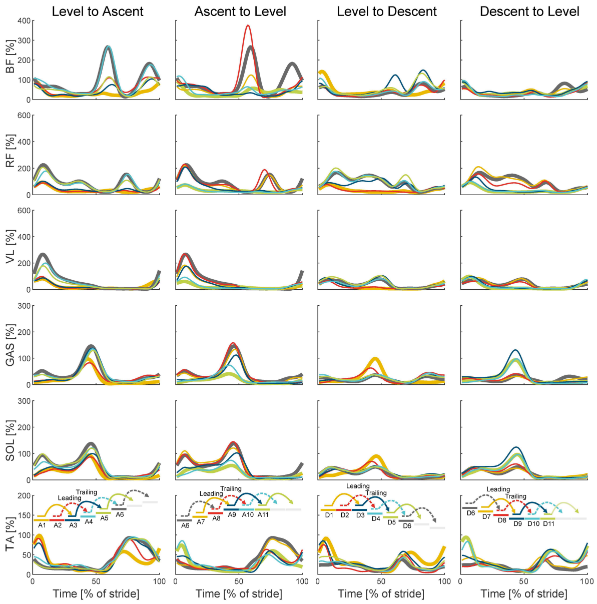

EMG for the low stair inclination. EMG for each of the six strides for the BF, RF, VL, GAS, SOL and TA for the stair transitions level to ascent, ascent to level, level to descent and descent to level.

S1 Fig.

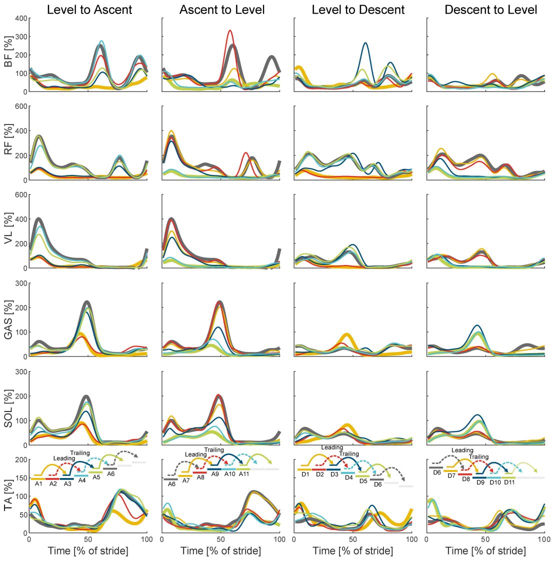

EMG for the normal stair inclination. EMG for each of the six strides for the BF, RF, VL, GAS, SOL and TA for the stair transitions level to ascent, ascent to level, level to descent and descent to level.

S2 Fig.

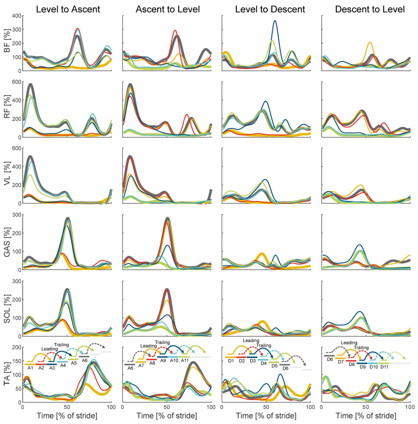

EMG for the high stair inclination. EMG for each of the six strides for the BF, RF, VL, GAS, SOL and TA for the stair transitions level to ascent, ascent to level, level to descent and descent to level.

S3 Fig.

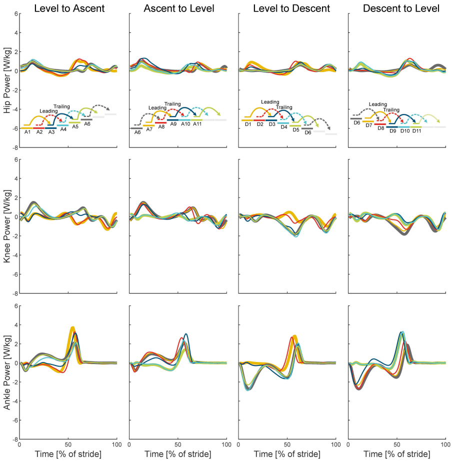

Joint power for the low stair inclination. Hip, knee and ankle power for each of the six strides for the stair transitions level to ascent, ascent to level, level to descent and descent to level.

S4 Fig.

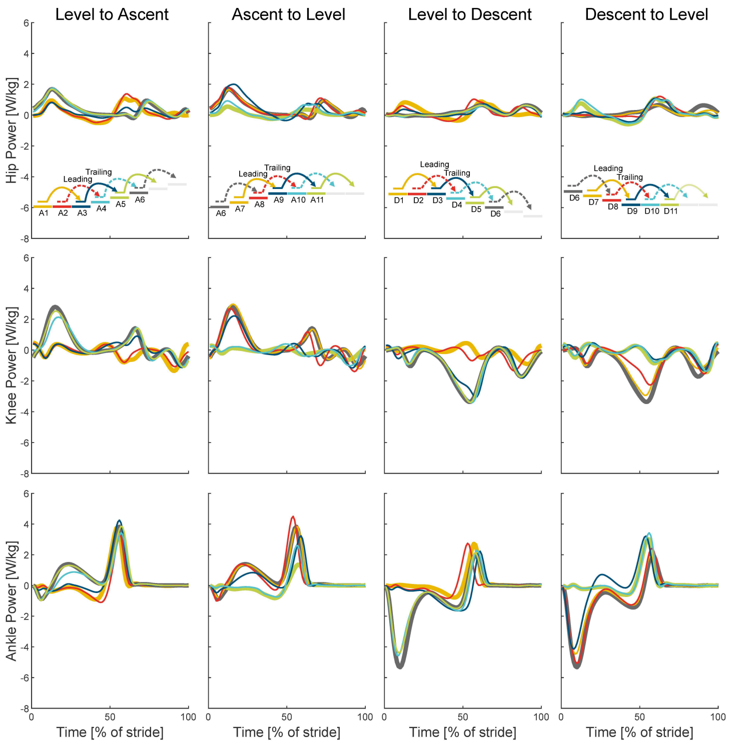

Joint power for the normal stair inclination. Hip, knee and ankle power for each of the six strides for the stair transitions level to ascent, ascent to level, level to descent and descent to level.

S5 Fig.

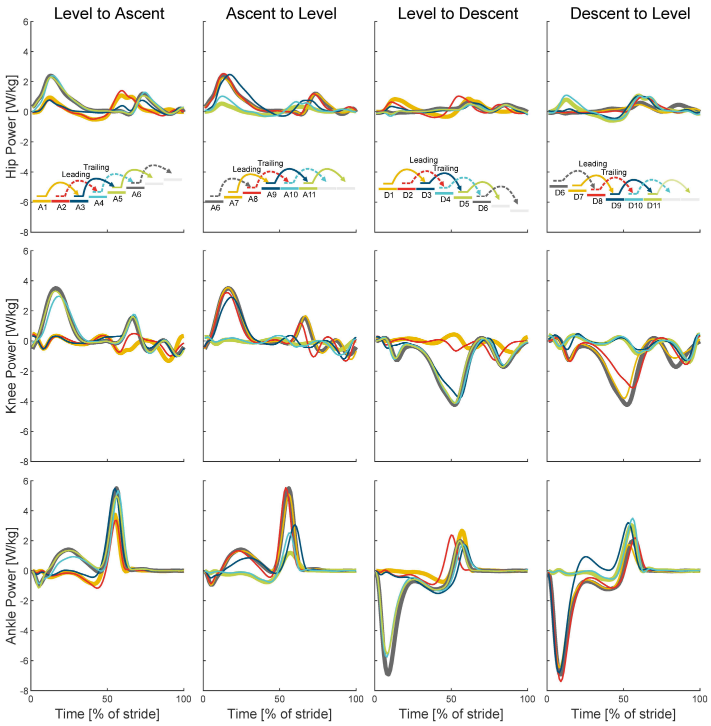

Joint power for the high stair inclination. Hip, knee and ankle power for each of the six strides for the stair transitions level to ascent, ascent to level, level to descent and descent to level.

S6 Fig.
